# Methods for Facial Expression Recognition with Applications in Challenging Situations

**DOI:** 10.1155/2022/9261438

**Published:** 2022-05-25

**Authors:** Anil Audumbar Pise, Mejdal A. Alqahtani, Priti Verma, Purushothama K, Dimitrios A. Karras, Prathibha S, Awal Halifa

**Affiliations:** ^1^Computer Science and Applied Mathematics University of the Witwatersrand Johannesburg, Johannesburg, South Africa; ^2^Department of Sustainable Engineering, Saveetha School of Engineering, Saveetha Institute of Medical and Technical Sciences, Saveetha University, Saveetha Nagar, Thandalam, Chennai 602105, Tamilnadu, India; ^3^Department of Industrial Engineering, King Saud University, Riyadh, Saudi Arabia; ^4^School of Business Studies, Sharda University, Greater Noida, India; ^5^Department of Computer Science and Engineering, Shri Venkateswara College of Engineering, Vidya Nagara Airport Road, Bangalore, India; ^6^National and Kapodistrian,University of Athens (NKUA), School of Science Department General, Athens, Greece; ^7^Department of Electronics and Communication, Government Engineering College Ramanagara, Ramanagara, India; ^8^Kwame Nkrumah University of Science and Technology, Kumasi, Ghana

## Abstract

In the last few years, a great deal of interesting research has been achieved on automatic facial emotion recognition (FER). FER has been used in a number of ways to make human-machine interactions better, including human center computing and the new trends of emotional artificial intelligence (EAI). Researchers in the EAI field aim to make computers better at predicting and analyzing the facial expressions and behavior of human under different scenarios and cases. Deep learning has had the greatest influence on such a field since neural networks have evolved significantly in recent years, and accordingly, different architectures are being developed to solve more and more difficult problems. This article will address the latest advances in computational intelligence-related automated emotion recognition using recent deep learning models. We show that both deep learning-based FER and models that use architecture-related methods, such as databases, can collaborate well in delivering highly accurate results.

## 1. Introduction

A human face has significant and distinguishing characteristics that aid in the recognition of facial expressions. FER is defined as a change in facial expression caused by an individual's internal emotional state. It is used in a wide range of human-computer interaction (HCI) applications, such as face image processing, facial video surveillance, and facial animation, as well as in the fields of computer vision, digital image processing, and artificial intelligence. Automatic facial expression recognition is a difficult topic that has piqued the interest of many researchers in recent years. In FER, the stage of feature extraction is critical. Alek et al. [1] demonstrated in the literature that facial expression accounts for 55% of total transmission while vocal and spoken communication contributes 38% and 7%, respectively.

There are two primary techniques for designing a FER system. As an initial step, some systems employ a sequence of images ranging from a neutral face to the peak level of emotions. In comparison, some systems use a single image of the face to recognize related emotions, and because they have access to less information, they often perform worse than leading approaches [2,3]. Apart from the approach type modeled by a FER system, another classification is based on the type of features employed in the recognition process, with a FER system utilizing one or both of these feature categories. The first set of traits is obtained from the facial organs' posture and the skin's texture. The second type of feature is geometric features, which hold information about various positions and points on the face and are used to analyze a static image or a sequence of photos by utilizing the movement of the positions and points within the sequence. Using face landmarks as a starting point for extracting geometric features is one way. Landmarks are significant places on the face that provide useful information for facial analysis. Numerous studies have been undertaken on the subject of facial landmark identification; however, they are outside the scope of this work. This work employs the *Python* module dlib to detect these points [4].

Two different things, which are involved in automatically identifying human emotions and psychology, are part of artificial intelligence. The big question that researchers are attempting to answer in the area of psychology and artificial intelligence is the identification of emotions. One of them includes both topics such as mood and accent, which is generated in both vocal and nonverbal sensors such as the tonality and aural alterations [5] which are widely accessible, for instance [6], and a quick mood assessment can be obtained, as well as other sources [7]. Results from Mehrian's study [8] demonstrated that 55% of information was sensory (emotional and verbal), with the remaining 7% being percent of having an unspecified physical component. The first indication a person gives of their emotional state being in a state is facial expressions, so many researchers are very interested in this modality.

First working in the extraction feature space to add new features to an existing representation can be a good thing because it will help the other features as well. Ekman and Friesen [9] noted that the Facial Action Coding System (FACS) and facial movement action units (AUs) assume that each coded movement in FACS involves at least one facial muscle. Ekman and Friesen first recognized that FACS facial movement, in FACS facial AUs, is utilized in such a way that face muscles and facial muscles are coded for each of their head movements (between several different individuals and/or races of subjects).

### 1.1. Motivation of This Study

Humans have a basic set of emotions, which are communicated via universal and basic facial expressions. Automatic emotion identification in images and videos will be possible if an algorithm that identifies, extracts, and assesses these facial expressions in real time is developed. In a social setting, facial expressions are powerful tools for communicating personal feelings and intentions. They play an important role in human social interaction. Face perception in the context of the situations in which they are seen offers important contextual information for facial expression processing. The ability to detect and communicate human emotions is critical in interpersonal relationships. Since the beginning of time, automatic emotion detection has been a controversial scientific subject. As a consequence, significant progress has been made in this area. Emotions are expressed via a number of means, including words, hand and body movements, and facial expressions. As a result, the ability to extract and comprehend emotion is critical for effective human-machine communication.

### 1.2. Contribution of This Study

1. Artificial neural networks (ANNs), backpropagation, multilayer perceptron (MLP), support vector machine (SVM), convolutional neural network (CNN), random forest, RNN, deep belief neural networks (DBNN), genetic algorithm, long short-term memory (LSTM), ResNet, SqueezeNet, SqueezeNet-TRN, and ImageNet are among the methods and techniques covered in detail
2. The many articles written for the research and published in the bibliographies have also highlighted the feature selection methods utilized in the different categorization methods
3. In addition, data with different degrees of precision have been given in order to evaluate the potential of the best approach
4. Furthermore, the details of the data gathering, which includes a variety of facial images, have been revealed
5. There is also a summary of the many facial emotions that have been classified throughout time by various researchers
6. A collection of literature on facial expression detection using the image and video classification has been researched and collected in order to provide unified knowledge on a single platform for new researchers interested in this area
7. Furthermore, the limitations of each study have been recognized and addressed in order to offer a basis for future research

In this paper, we examine the current frontiers of emotion detection through different architectures with regard to the full range of expressive modes, given facial cues. The recent results from 2016 to 2021 are reported along with an analysis of the most prevalent problems and recent contributions to resolving them. It is set up in the following manner.  Section 2 begins with the basic types to describe facial expressions such as FACS and prototypic emotional expressions.  Section 3 represents face detection and emotion recognition system structure.  Section 4 focuses on recent FER findings with real-time applications.  Section 5 gives a short summary of FER challenges in the area and speculations on what lies ahead.  Section 6 presents links to some public databases used in FER tasks followed by basic types of emotion recognition in Section 7. Deep learning-based facial emotion recognition is briefly explained in Section 8.  Section 9 represents a comparative analysis and discussion on FER. Finally, we end with an outlook on what the future will hold with the conclusion.

## 2. Basic Types to Describe Facial Expressions

When it comes to describing facial expressions, there are two main methods to consider.

### 2.1. Facial Action Coding System

**Figure 1:**
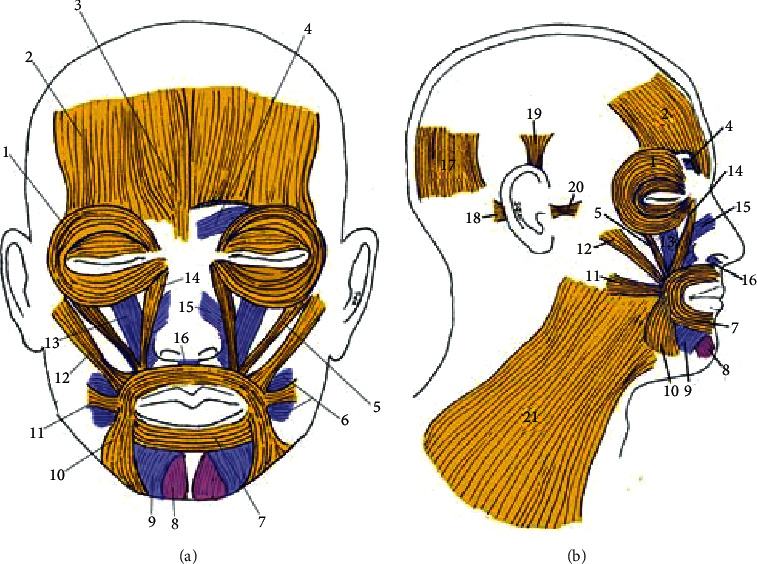
Muscles of facial expression [10].

**Figure 2:**
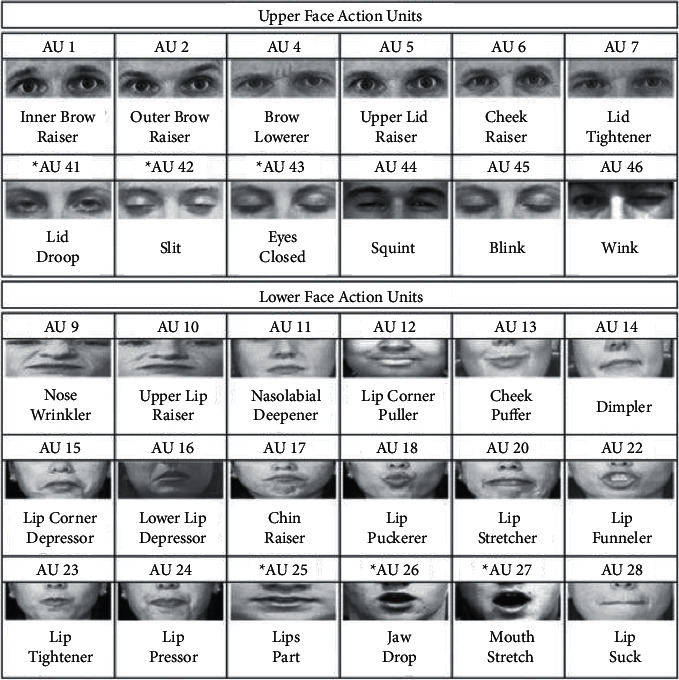
Facs action units [12].

The FACS [11] identifies little changes in facial characteristics. This widely used method in psychology is based on a human observer's observation and consists of 44 action units connected to the tightening of groups of facial muscles in order to detect facial emotions (see Figure 1; muscles of facial expression are 1. frontalis; 2. orbicularis oculi; 3. zygomaticus major; 4. risorius; 5. platysma; 6. depressor anguli oris). In addition, a couple of the action units are shown in Figure 2. FACS is often coded and labeled manually by skilled individuals who examine slow-motion video footage of face muscle contractions before coding and labeling them. In recent years, many efforts to automate this procedure have been undertaken [13]. The system's dependence on descriptive data labels rather than inferential data labels, on the other hand, may provide a difficulty since it allows for the capture of nuanced facial expressions, among other things. To estimate emotions using FACS data, the FACS data must first be transformed into a system capable of doing so. This kind of behavior is modeled by the Emotional Facial Action System (EMFACS) [14].

### 2.2. Prototypic Emotional Expressions

**Figure 3:**
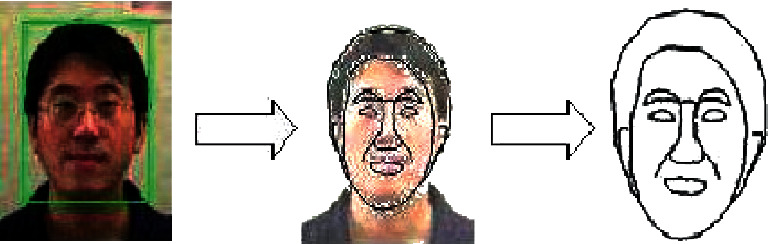
Face detection and alignment processes [16].

The majority of FER systems do not define face characteristics in depth; instead, they begin with prototype expressions. The human universal facial expression of emotion set, which includes six kinds of fundamental emotions [15], is the most frequently used collection of prototype facial emotion expressions. It is the most often used set of facial expression prototypes. These fundamental terms are utilized because they are cross-ethnic and cultural barriers (Figure 3). This indicates that these emotions exist in everyone and may be seen in a range of situations [17]. They include fear, anger, joy, sadness, disgust, surprise, and a neutral remark. This system may be used in two ways: as a traditional classifier to identify the emotion of the person shown in the image, or as a probabilistic estimator to estimate the likelihood of the person displaying emotion in the image. In the second instance, it performs the function of a fuzzy classifier.

## 3. Face Detection and Emotion Recognition System Structure

FER may be utilized as a standalone facial recognition system or as a module inside an existing facial recognition system. As a result, it is prudent to investigate the overall design of the system. In general, the system is made up of four components, as shown in Figure 3. The face detection component is responsible for detecting whether or not a face is present in the input media.

If the input medium is video, face recognition is only done on crucial frames, with the other frames monitored using a tracking technique. This is done to enhance the system's overall resilience. Face alignment, on the other hand, is comparable to face detection in that it gives a more precise position of the identified face. This phase involves identifying face features such as a person's nose, eyes, and brows, as well as other facial features. The image is then subjected to a technique known as geometric normalization, which alters photometric characteristics such as brightness and contrast. Then, feature extraction is utilized to categorize labels like gender, identity, or expression. The extracted feature may be sent into a classifier or compared to training data, depending on the conditions.

The components of face detection, face alignment, and feature extraction are briefly explained in Sections 3.1, 3.2, and 3.3, respectively.

### 3.1. Face Detection

Face detection is the initial stage in the face recognition process and is essential to the system's overall effectiveness [14]. Faces in films may be recognized using visual cues such as facial expression, skin tone, or movement in the film. Numerous effective methods are restricted to improving the appearance of the face [18]. This may be because these algorithms avoid the challenges associated with representing 3D structures like faces. The face/nonface border, on the other hand, may be extremely complicated, and it is necessary to use 3D variations in order to identify facial emotions. As a consequence, many solutions to this issue have been proposed since the 1990s [19].

Kenli and Ai [20] created a detection technique that uses Eigen decomposition to find abnormalities. They combine a generic face with a range of “eigenfaces”. The researchers [21] distinguished this from Sung and Poggio, who focused only on ‘eigenfaces'. They did, however, use Bayes' rule on nonfaces to determine the likelihood of occurrence. Rowley et al. [22] utilized neural networks to distinguish between images with and without faces, while Osuna et al. [23] trained a Kernel support vector machine to distinguish between images with and without faces. To retrain the SVM, a bootstrap approach was employed, and the results were encouraging.

Additionally, Schneiderman and Kanade [24] used AdaBoost to build a classifier based on a picture's wavelet shape. As a consequence, the method requires a significant amount of computing time. Viola and Jones [25] overcame this problem by substituting Haar features [26] for the wavelets. Haar features were computationally less expensive than wavelets. This is the first demonstration of a real-time frontal view facial recognition system [27].

Several enhancements to Viola's framework have been proposed. The Haar characteristics were rotated in-plane by Lienhart and colleagues [28]. Li et al. [29,30] suggested using a detector pyramid to cope with out-of-plane rotation, and this system may be utilized for multiview face recognition as well. Eigenface and AdaBoost were introduced as techniques for facial detection. Eigenface is considered the most simple technique for face detection, whereas AdaBoost is regarded as the most successful. AdaBoost may also be used to extract face characteristics.

### 3.2. Face Alignment

Face alignment, which involves the detection of facial feature points, may result in more accurate face localization when performed in conjunction with face localization. A comparison of face recognition algorithms and facial alignment methods is shown in Figure 3. Face detection, as illustrated in the picture, assesses regions of an image, while facial alignment is accurate to the pixel level.

Various methods to address this problem have been suggested since the 1990s. Gu et al. [31] used histograms to identify the corners of the mouth and eyes in a picture. Marian and colleagues [32] used Gabor filters in images to identify the medial cleft and pupils. Despite the fact that many methods have been tried, the Active Shape Model [33] is the most effective of the curve fitting algorithms currently available.

Cootes et al. [33–35] proposed the Active Shape Model (ASM) for usage with images that include faces. The ASM's durability, speed, and accuracy have all increased considerably since then. Li et al. [36, 37] created the Direct Appearance Model by coupling Gabor filters with ASM, which was subsequently validated by other studies. Other authors [38] have enhanced ASM for local searches by including 2D local textures in the search results.

### 3.3. Feature Extraction

The feature extraction technique transforms pixel data into higher-level representations of the face in the image, such as texture, color, motion, contours, and the spatial arrangement of the face. This collected data is then used to assist in the detection of trends throughout future categorization procedures. During the feature extraction procedure, the dimensions of the input space are typically decreased. It is critical to retain information with high stability and discrimination while also maintaining a high level of stability during this process. To identify a person's face, a number of unique characteristics are utilized [39].

Recently, the coefficients of eigenface have been used as features; [40] used an extension of eigenface, called Tensorface, which has shown promise. The Active Appearance Model [41] deconstructs the image of the face into “shape” and “texture”. The shape vector refers to the contours of the face, while the texture vector refers to the “shape-free” textures of the face. Potential Net was used by Matsuno et al. [42] to extract features with a two-dimensional mesh. The above-mentioned methods are seen as holistic features, because they look at the overall structure of an image. Another type of feature that only focuses on small regions is called local features. Local features can be used directly as image subwindows as in the case of Colmenarez et al. [43]. They used nine subwindows positioned around the facial features. Gabor Filter, a popular wavelet filter, has also been used [44, 45] and has had reasonable success regarding the visualization in the primary visual cortex. Primitive topographic features have also been used by Yin and Wei [46] to represent faces. Yu and Bhanu [47] utilized an evolutionary algorithm to automatically generate features instead of defining the features beforehand. In video-based FER, the dynamic changes of expression can also be seen as a feature. The Geometric Deformation Feature that was proposed by [48] has the ability to geometrically displace landmarked nodes. Facial Animation Parameters as used by Aleksic and Katsaggelos [1] are based on the Active Shape Model.

### 3.4. Emotion Classification

The automatic expression recognition problem has received a lot of attention with a variety of classifiers being applied to it. One solution, offered by Matsuno et al. [49], looked at the threshold of normalized Euclidean distance between features to categorize a facial expression. Another solution [43] makes use of Bayesian recognition to find facial expressions, which maximizes the likelihood of the image. Other methods include Locally Linear Embedding [50], Fisher discrimination analysis [51], and Higher-Order Singular Value Decomposition [52] to name only a few [1,53,54]. Currently, support vector machines [55–57] and neural networks [58–61] offer the best accuracy and are seen as most successful when applied to the automatic expression recognition problem.

## 4. Real-Time Applications of FER

**Figure 4:**
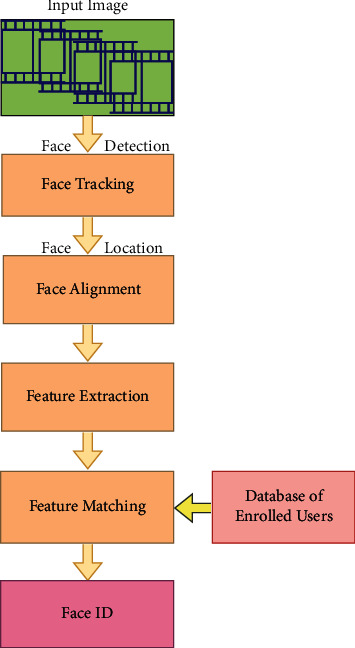
Face recognition processing flow.

It is only in the emotional mechanisms that a lack of progress was recently discovered [62]. It has been found that emotional mechanisms take precedence over rational processes in the brain, which can be seen as either being advantageous or detrimental depending on their presence or absence [63]. Worse feelings give rise to negative thoughts, which tend to dampen a person's creativity when looking for solutions to the challenges at hand and lead to them getting you into deeper trouble. It has been found that states such as anger, sadness, fear, and happiness each have their own distinct patterns of blood flow to the brain and have an influence on that of mood [64]. A great deal of research has demonstrated that positive emotions like joy, acceptance, trust, and satisfaction can assist learning, while negative emotions can bring about learning disabilities and affect the process. Anxiety and depression can hinder memorization in various ways. These states can show up in different ways, such as causing stress, which increases with despair, and leads to increased feelings of anger and fear, or fear, or stress itself may lead to worse than depression. When it is difficult for a student to acquire information, intelligent feedback can help them overcome their lack of motivation. For the latter, the computer should be able to know what learners are feeling, give learners opportunities to expand on their understanding, handle their interests, and provide them with pertinent information and timely feedback [8].  Figure 4 depicts end-to-end face recognition processing flow.

According to [65], enabling students to provide students with affective and intelligent feedback in an e-learning system, for instance, involves the use of virtual persons, namely, embodied conversational agents (or other virtual agents that are capable of communicating both verbally and nonverbally, such as animated graphical characters) that can convey emotions or other sentiments and provide information to them using body language. It is much more effective in having a machine that talks to the user but does not take any of his or her reactions into consideration. Such systems may be easier said than done, but an immense challenge exists in implementing them, given our currently crude ability to recognize human feelings and behavior.

In MobileNets, they developed a set of efficient convolutional neural models, Andrew G. Howard et al. [66]. A class of efficient MobileNets convolution models were developed by him, as part of a class called MobileNets. The neural architecture has been designed with a novel type of convolution called depth separable and is made up of factors, instead of connected layers, of depthwise convolutions. Depthwise convolutions are two layers: they are separable, and the first is a depthwise layer consisting of two separable convolutions. According to normal convolution, it is over 10 times more efficient. However, it only applies lowpass and highpass effects to the signal; it does not add any new features. To do this, they did the addition of other operations such as pointwise convolution (e.g., which does the sum of pointwise convolution outputs) and implemented 1x pointwise convolution, respectively. By adopting two additional hyperparameters, they try to increase their efficiency. Thus, the network can be made thinner by the increase in network width by a multiplicative factor *α* and resolution by a nonuniform *ρ*, and cost reduction can be done at each layer. Different hyperparameters allow the model builder to choose a model that will have just the right number of parameters for their own application, but without error. Various methods and functions are demonstrated by this model with different examples, with facial features as well as the measurement of object dimensionality.

Recognizing emotions is difficult because they are ambiguous and therefore prone to error, but in many cases, there are various things that can be used to discover them. Ekman claims that there are eleven basic emotions found in human facial expressions which can be classified into seven groups: happiness, anger, sadness, fear, disgust, surprise, and contempt [67]. This after the turn of the millennium got a boost from successful experiments in face recognition and audio-visual media and has paved the way for further research on automatic affect recognition of effect. Face expression popularly suggests that recognition of emotions is simply a method to look for patterns that indicate whether or not a person is empathetic popularly known as “to judge by the look on their face”. The FACS is used to code different types of facial actions, which include facial motions, and numerous AUs are created; each one of these classes contains unique entities; finally, emotions are determined by AU designations.

Bartic and colback have done extensive research on emotional recognition results, which is accurate and comprehensive, as Bartlett and Mattivi [68] reported. Features that are based on geometry (e.g., the shape of the eye, or the angle of the eyebrows) measure the face's facial elements such as the length of the nose or the width of the mouth. Empirical approaches use different machine learning algorithms to analyze the detected face, all of which are capable of determining features, with the goal of classifying it into an emotional state. At this time, it is possible to recognize emotions using facial expressions using the information from the applications that were developed by [69]. Six basic emotions are identified by the facial analysis software tool known as the Face Reader, with an accuracy of 89%. In the work referenced in [70], facial recognition researchers have done significant progress by leveraging local characteristics in a database of people. A face can reveal both stress levels as well as the degree of emotional interest as well as being energetic endurance, which is something that is not widely understood.

There is also significant information that can be gleaned from gestures and postures in regard to an individual's emotional state and attentive state. This research is lacking; however, these topics have not been extensively studied. When applied to the analysis of the user's previous and current interactions with the web data, information from the previously stated sources can help prove the user's current cognitive state [71]. Generally speaking, the development of an affective guidance system depends on emotional state and entertainment in addition to the study of the various kinds of influencing factors, with the result that feedback is appropriate. Robotic tutors are expected to offer virtual classes in a redesigned, so they can better reflect their pupils' personalities and respond to pupils' emotional states. The researchers in [72] contend that the presence of frustration, boredom, motivation, and confidence is equally essential for a computer tutor, and they conduct an analysis of each method of feedback they have used to gauge the other. Basic emotions like fear, sadness, and happiness are found in [73] articles by the authors. A style of ECAs known as “expansive empathy” is performed before performing “reactive empathy” which is then presented with expression and voice.

Although studies have demonstrated that automatic expansion is the most difficult; here, the authors show different views of the complexity of expanding every case individually (possible), a complexity scale, formed by Philipp et al. [9] that shows different techniques in use. The critical variables to consider when using this technique include head pose, skin condition, and/age, which may vary depending on when and if one is taking photos in high light versus low light conditions, as well as their position, and additionally the issue of occlusion created by the scarf or other illumination. Several techniques are used for the extraction of facial features, for example, geometric features, and texture features, for example, LBP [74] and Gaborlet unit activity, while the Generalized Local Binary Pattern Classification (LBC) and Directionalized Gabor (GDA) are used to extract facial landmarks [75]. Since it has started being widely used in recent years, mainly due to the application of convolutional neural networks and recurrent neural networks, it has proven to be a very successful and efficient technique for emotion recognition. Several neural networks have been developed in this area to help with the development of deep architectures, all of which produce commendable results [76].

## 5. Current Problems/Challenges in Face Detection and Emotion Recognition

This section explores cutting-edge methods for analyzing and understanding facial expressions. The following essential issues must be solved while designing a facial emotion recognition system: face detection and alignment, normalization of the facial image, extraction of critical attributes, and, finally, classification. Currently, the majority of systems conduct these processes sequentially and independently. As a result, this section will first discuss the problems associated with identifying facial emotions, followed by an examination of how the aforementioned processes have been handled in various studies.

However, distinguishing the features of a human face and interpreting its emotional state are both difficult undertakings. The fundamental problem originates from the nonuniformity of the human face, as well as additional restrictions connected with lighting, shadows, facial location, and orientation concerns in various circumstances [77]. Humans are born with the ability to perceive and grasp facial expressions and emotions with little or no effort; nevertheless, computer systems continue to face significant hurdles in recognizing effective and robust facial expressions. Numerous deep learning techniques, including multilayer perceptron (MLP) neural networks and support vector machines, have been studied as a family of techniques for improving the robustness and performance of fundamental machine learning classification techniques, such as MLP neural networks. To be effective, human behavior analysis must be adaptable to a wide range of contexts. Deep learning algorithms may be able to deliver the required robustness and scalability on new types of data.

The parts that follow will go through the most important challenges in automated facial expression recognition in detail. In this situation, acquiring task-representative data, overcoming ground truth collection challenges, dealing with occlusions, and modeling dynamics are all key difficulties.

**Figure 5:**
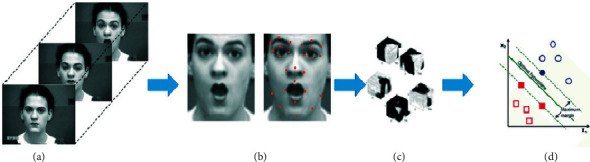
Conventional FER method [28]. (a) Input images. (b) Face detection and landmark detection. (c) Feature extraction. (d) FE classification.

Procedures utilized in standard FER techniques are depicted in Figure 5: face region and facial landmarks are detected in input images, spatial and temporal features are extracted from the face components and landmarks, and facial expression is determined using pretrained pattern classifiers based on one of the facial categories (face images are taken from the CK + dataset [78]).

## 6. Databases Used for Facial Emotion Recognition

Facial recognition is getting better and more prevalent each year, and consequently, facial databases have expanded tremendously [79]. When modeling of recognition requires visual or audio examples, you have to give, model enhancement or training of the model requires a database of those kinds, and this, as well as class labels for them, which gets progressively larger and larger as the number of examples increases, expand is required [80]. For example, there are various possible applications for emotional recognition, ranging from simple human-robot collaboration [81] to being used to identify people suffering from depression to serving as a depression detector [82].

Although the algorithm most commonly accepts image/portrait datasets, which are uniformly lit and fixed in position, this form, in the top portion, an alternative version is one where the top and bottom halves are aligned but cropped differently. To be able to compare to the pixelated versions, the NIST mugshot database [83] also offers a clear, grayscale option for finding image IDs of 1573 individuals on a neutral background. But it also takes the authors to go out into the real world to realize how light conditions and occlusions work in the context of real life in order to comprehend the situations [84]. Using this method [85], the subjects were easily rotated and the effects of different lighting on their appearance were studied in the M2VTS database which features the faces of 37 subjects in a variety of rotated and lit positions. The emotions included in a database define its function. Many databases, like CK, MMI, eNTERFACE, and NVIE, opt to record the six basic emotion categories proposed by Ekman. Many databases, like the SMO, AAI, and ISL meeting corpus, try to classify or contain general positive and negative emotions. Some try to evaluate deception and honesty, such as the CSC corpus database. The most well-known 3D datasets are BU-3DFE, BU-4DFE, Bosphorus, and BP4D. BU-3DFE and BU-4DFE both have six-expression posed datasets, with the latter having a higher resolution. Bosphorus tries to address the issue of having a wider range of facial expressions, while BP4D is the only one of the four that uses induced rather than posed emotions. The main benefit of deep learning is that it opens the neural networks to various databases, allowing them to grow with the addition of a wide range of new inputs, examples, facial expressions, and constant changes in expressions.

## 7. Facial Expression, Speech Emotion, and Multimodal Emotion Recognition

We have covered the three main types of emotion recognition techniques in this section: facial expression recognition, speech emotion recognition, and multimodal emotion recognition utilizing visual representations. We have also spoken about how these methods may be used in a variety of situations.

### 7.1. Facial Expression Recognition

  Facial movements are essential in nonverbal communication for expressing emotions. Facial expression recognition is important in a broad range of applications, including human-computer interaction and health care. Mehrabian discovered that 7% of information is conveyed between people via writing, 38% through conversation, and 55% through facial expression. Ekman and colleagues [86] defined six basic emotions: pleasure, sadness, surprise, fear, and anger. He demonstrated that people, regardless of culture, have these emotions. Feldman et al. [32] propose that emotions may be represented using two orthogonal dimensions: valence and arousal. He observed that everyone displays their feelings in a unique way.

Furthermore, when people are asked to express their feelings on a regular basis, their responses vary greatly [87]. Arousal levels vary from calm to eager, while valences range from positive to negative [88]. This research would categorize the information based on valence and arousal changes. Researchers first developed methods for manually extracting facial expressions by developing algorithms for extracted functions such as the Gabor wavelet, the Weber Local Descriptor (WLD), the Local Binary Pattern (LBP), and multifeature fusion. These properties are susceptible to topic imbalances and may result in a substantial loss of texture information from the original image. The use of deep neural network models to face expression analysis is now the most popular subject in facial recognition. Furthermore, FER provides a wide range of social life applications, such as intelligent protection, deception detection, and intelligent medical practice. The authors of [89] discussed facial expression recognition models developed using deep learning methods such as DBN, deep CNN, and long short-term memory (LSTM) [90], as well as their combination.

### 7.2. Speech Emotion Recognition

**Figure 6:**
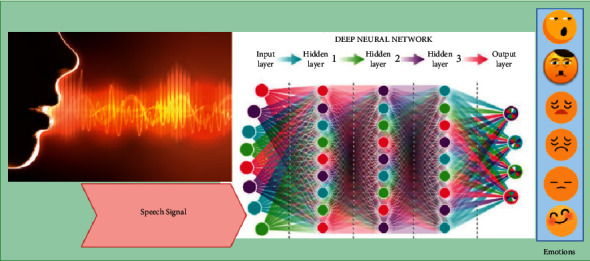
Speech emotion recognition [8].

Speech recognition is a key component of human-computer interaction systems. They will communicate their feelings via their words and facial expressions. Emotions are often identified using speech recognition algorithms [91]. The early attempts at emotion detection in a speech focused on categorizing speech by extracting artificial features. Liscombe et al. (2003) looked at the relationship between various emotions and a set of continuous speech parameters based on basic pitch, amplitude, and spectral tilt. Throughout the years, many algorithms for detecting emotions in human speech have been created [92]. Many machine learning techniques have been proposed, including support vector machines, hidden Markov models, and Gaussian mixture models. Deep learning has been widely used in a wide range of speech domains, most notably voice recognition [93]. Convolutional neural networks have also been used to detect emotions in speech; they show that bidirectional multimodal emotion recognitional RNNs (Bi-LSTM) are more successful at extracting important speech characteristics, thereby increasing speech recognition performance [1].  Figure 6 illustrates the end-to-end “Speech Emotion Recognition” system.

### 7.3. Multimodal Emotion Recognition

In research, multimodal emotion processing is still extensively utilized. Through the utilization of new study modalities, this expansion would assist in a better understanding of emotions (video, audio, sensor data, etc.). To achieve the study's goal, a variety of techniques and tactics are used. Many of them use big data techniques, semantics, and deep learning. Emotions are complicated psychophysiological processes that occur nonverbally, making identification difficult. Multimodal learning is much more effective than unimodal learning [94].

**Figure 7:**
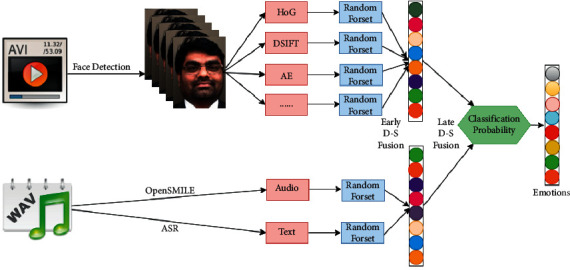
The framework for multimodal emotion recognition NN [94].

There is a basis for a neural network for multimodal emotion recognition, with a focus on visual input in recognized faces. Their approach was inspired by the winners of the 2013 and 2014 EmotiW challenges. Chen et al. [95] proposed their approach in answer to a multimodal emotion detection issue (MEC 2016). This technique retrieves multimodal features in order to determine the emotion of the character in the video. Among them, the facial CNN feature has the highest discriminative power for emotion recognition. Previously [96], we retrieved several features using both traditional and deep convolutional neural network (DCNN) methods. On testing sets, this approach delivers an exceptionally promising result. We describe the techniques used to generate the team submissions for Beijing Normal University's 2017 Multimodal Emotion Recognition Challenge (MEC 2017). Many features were retrieved, including an autoencoder (AE), a CNN, a dense SIFT, and an audio feature, and a Dempster-Shafer theory fusion method was provided for merging different prediction results based on these features. The framework for multimodal emotion recognition NN is shown in Figure 7.

Furthermore, research has tried to combine data from different modalities, such as facial expressions and audio, audio and written text, physiological signals, and various combinations of these modalities [97]. This technique is currently being enhanced to increase the accuracy of emotion detection. A multimodal fusion model may generate emotion detection results by integrating physiological data in a number of ways. Recent advancements in deep learning (DL) architectures have made it possible to use deep learning for multimodal emotion recognition. The deep belief network, the deep convolutional neural network, the LSTM [55], the support vector machine (SVM) [98], and their combinations are all deep learning techniques.

## 8. Deep Learning-Based Facial Emotion Recognition

**Figure 8:**
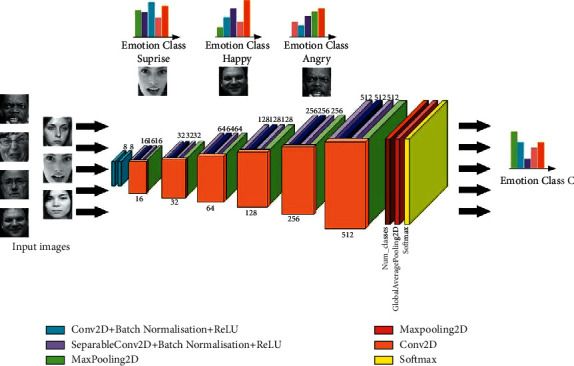
Deep convolution neural networks based on FER [99].

Deep learning algorithms have recently emerged as a viable replacement to traditional feature design techniques since they provide automated feature learning instantly. Deep learning research may lead to better representations and the creation of new models for learning these representations from unlabeled data. Because of the introduction of powerful GPU processors that allow high-performance numerical computing in graphics cards, these techniques have become computationally feasible. Deep learning techniques such as convolutional neural networks, deep Boltzmann machines, deep belief networks, and stacked autoencoders are used in practical applications such as pattern analysis, audio recognition, computer vision, and image recognition, producing impressive results on a variety of tasks. Li et al. [100] provided a thorough evaluation of the aforementioned DL techniques adapted to the FER issue lately. Ginne et al. [5] have given an overview of CNN-based FER techniques. Deep convolution has been used extensively in FER research. The focus of network research has been on improving expression recognition accuracy. It is important to observe how, at the end of the day, a smaller CNN architecture with the same level of accuracy is feasible. Deep convolution neural networks based on FER are depicted in Figure 8 providing more effective scattered training, as well as a more controllable parameter model, as well as improved deployment suitability on memory-constrained devices, reducing costs and allowing for greater distribution.

In the last decade, CNNs have done well for FER, as shown by their use in a number of cutting-edge algorithms. Many FER competitions [101], including the previous year's EmotiW challenge, were won by a kind of CNN architecture with few layers. Facial emotion recognition has served the public well for decades prior to the field of deep learning breaking, and a group of brilliant researchers has tried to stay abreast of the current research efforts in that field, while others have undertaken to learn from its methods and discoveries. In recent times, many researchers offered novel and recurring practices for applying deep learning in order to security problems in an effort to enhance detection. Validation users currently do additional validation on a number of static or sequential databases before allowing their information to be used in a live database.

The VGG-16 model (developed by the University of Oxford's Visual Geometry Group (VGG)) may be considered a watershed moment in the history of deep CNN models [102]. It was pretrained using the ImageNet database [103] to extract features from images that might be used to distinguish between image classes. Numerous recent studies show that VGG-16 performs well on image recognition and classification datasets from a variety of fields.

Marco et al. [104] proposed Deep Convolution Neural Networks (DCNNs) which are used in the cross-database search. After that, facial images had to be reduced to 48 × 48 pixels; the rest of the same pictures had to be searched for locations and landmarks to be extracted. Finally, they had augmented the database with additional data, and only then did they were able to create it. Subsequently, the data moves on to two classification stages where the softmax (SF) is expanded and fed into the fully connected softmax (XF) network after the first classification stage. To avoid overfitting, they suggest using local CNNs in combination with convolutional layers that are fine-tuned for specific use cases.

In [105], the authors have shown that the results prior to training were used to discover how to influence the final outcome When it expanded, the first CNN expansion, when it lowered the size to 32 × 32 and also used data normalization with 8-connected pools followed by downsampling (normalization of 32 × 32 to a 256 final dimension), and when that was done, cropping was employed. Gaining the most mass is something that happens only at the competition, so the athletes who have gained the most muscle will play in the games. The information used this third party search tool to assemble a total of three transparently accessible databases: the CK+ and JAFFE, as well as the BU3DF. One also discovers a wide range of beneficial practices when considering these studies, such as utilizing all of these techniques and products together; studies show the difference between these things yielding different results.

The preprocessing techniques employed by Anil and his associates [8] have also been applied in the study by the authors. They are devising a new CNN face recognition algorithm for people who have not yet been recognized. They have two convolutions allowing layers and a dropout layer that gives the net activation of one in order to predict more accurate results. They use maxing with one extra activation as well and a final convolution (expanding) layer in the last step to increase accuracy and flexibility.

An important concern raised by Cai et al. [106] deals with the fact that the closing or the disappearance of small-town public libraries is managed by solving CNN, which employs Sparsity Batch Normalization. Dropout may be added to network building to help against overfitting and SBP (Support, Gradient, and Regularization) as a second stage to improve model generalization capacity, with the property of being used in networks twice (as a support for and then starting with 2-convol reg and ending with SGD), to strengthen the network. Li et al. [107] proposed using a CNN to tackle the facial distortion problem, in particular; the authors are doing so by first extracting the data from the VGG network and then running the ACN. *Affect*. Also, this architecture has been and has already been employed in the Affect Net, the RAF database, and the FED-RO database.

Yolcu and his colleagues [108] proposed that faces may be the most important aspects to focus on where Y could be realized in order to accurately take and record a single facial feature as small as an eyebrow or on top of the nose, like the face; the three microscopes have to examine a three-millimeter area. Before using the image for registration, they crop the face to avoid blurring. They work with only key-point facial regions until they are finished using the CNN to ensure that registration has been completed. Prior to this, the project being filmed in full-frame, the subjects' faces were exposed to be greatly improved and details increased; for example, expressions were added to them in order to show more complexity. There are more proven benefits; for example, studies show that utilizing photographs is a better approach for capturing the true appearance of your screen targets.

To figure out the significance of the CNN attributes in FER2013, researchers investigated and added to the already discovered findings of Agrawal et al. [109] (this also included research on Agrawalwarsh et al.) in 2019. Beyond that is an image memory pool at 64 × 64 pixel resolution, the network will have a certain type of an allowable number of convolution layers, and ad hoc pooling will take the second position, followed by other admissible filters before classifiers. The results of the study demonstrate that determining models achieve a 61.23% and 63.77% of their accuracy using isolated units, compared to adjudging, or dropout models, but do not have well-connected layers.

New ideas were proposed by Deepak et al. [110], where they advise that residual blocks contain two channels, each of which has two consecutive convolution layers. These pretrained models after cropping and normalizing the images on the JAFP and CK + datasets before they go into training mode allow identifying and eliminating unwanted variations in intensity.

Kim et al. [118] compared the three emotional state models: they used CNN with LSTM to show facial expression variation in space and time. First, CNN expanded the facial state information into a spatial representation and used this representation to represent the temporal variation of facial information; afterward, CNN expanded temporal representations and preserved the spatial information. Furthermore, the authors [119] created a new network architecture known as Spatiotemporal Convolutional Network with Nested LSTM (STC-LSTM), which preserves both temporal and spatiotemporal features with a 3D-Cell T-LSTM style CNN.

In the facial expression sequence level, DCNN was postulated by Liang [111] consisting of two deep layers, one of which handles spatial features and the other temporal features, which are treated as features that are then merged and expanded into vectors of 256 dimensions to form the large facial emotion category vector; that is, the expression differentiated into six basic emotions is utilized. They went through the Multitask Cascade Computational Net for face detection, after which they broadened the database with the technique of data augmentation. It is based on all of the scientists' opinions about classifying the basic emotions that previously state that the emotion categories are happiness, fear, surprise, sadness, and neutral. Instead of presenting the theories and thoughts of other researchers, we exhibit different proposals from those who recommended.

## 9. Comparative Analysis and Discussion on FER

**Figure 9:**
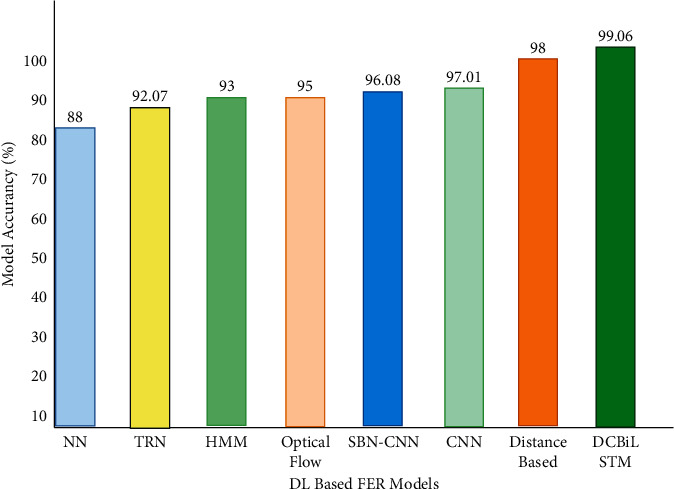
Deep learning-based FER models.

We made it clear in this paper that there is a strong demand for expanded research on the topic of FER far beyond shallow learning techniques. The full form of the automatic FER goes through four main data processing, a few proposed architectures, and finally getting to the main model's emotion recognition. Many preprocessing techniques were mentioned in this review, such as the cropping and resizing of images to speed up training and normalization, as well as the overfitting of data. Lopes et al. [120] have presented all these techniques well, according to Lopes and his colleagues.  Figure 9 illustrates deep learning-based FER models. The various methods and contributions presented in this review achieved high levels of accuracy. Moselhi et al. [121] demonstrated the key significance of the use of neural networks and connectivity expansion layers in neural network architectures. Like many before them, the authors Mohammadpour et al. [122] prefer to extract AU from the face, rather than do face-to-recognition first, first. This study is being conducted to determine whether occlusion images exist or not, as well as to try to gain greater insight into the network. Pise et al. [8] have examined the incorporation of the leftover blocks. While text images allow only for larger eyes and smaller faces, the addition of the iconized face to the network improves accuracy when using small images, as is demonstrated by Yolu and Ayiv [108]. Two-concept CNN architecture expansion was added after a long and thorough analysis of the recognition rate by offering two more feature articles to know the impact of CNN parameters. Favorable results have been observed in most of the methods attempted, which means more than 90% of these projects achieved some level of success. Researchers who study spatial and temporal features first provided several combinations: the combination of CNN-L and 3D-CNN is typically applied to give a boost to spatial features but boosts temporal features too. As can be demonstrated according to the work of Yu and his colleagues, the methods proposed by Kim et al. [118] and Liang et al. [111] provide a better level of precision than the one that was performed by the Kim group [118]. That comes out to an effective volume expansion factor of about 99%.

In CNN applications, both temporal and spatial networks have demonstrated their accuracy. That is why the researchers chose LSTM, which is effective for sequential data in general, but especially for time-dependent data in order to achieve high accuracy in FER. To date, CNN parametric modeling and the most difficult algorithms used by CNN researchers are softmax and Adam optimization. To validate the neural network architecture, we also tested the model across multiple databases, and our findings indicate that there are no significant differences in results.

Table 1 summarizes the arguments made previously, with particular emphasis on the architecture, database, and recognition rate discussed in the linked articles.

## 10. Conclusion

In this study, recent developments in FER were presented, and presented studies allowed us to track the latest developments in this area. Over the past year or so, a number of different researchers have devised different CNN architectures and some outside the lab produced reference databases. To facilitate an accurate emotional detection, we need to have provided previously obtained as well as experimental tables (spontaneous as well as lab) (emotion as reference). Also, we introduce a discussion which emphasizes the fact that machines are already able to recognize more complex emotions, implying that the emergence of human-machine collaboration will become more and more commonplace.

## 11. Future Work

While FER is an important source of information about an individual's emotional state, it is always limited by learning only the six basic emotions plus neutral. It is in conflict with what is present in everyday life, which contains more complex emotions. This will encourage researchers to expand their databases and develop powerful deep learning architectures capable of recognizing all basic and secondary emotions in the future. Additionally, emotion recognition has evolved from a unimodal analysis to a complex system multimodal analysis in the modern era. Leon et al. in [123] demonstrate that multimodality is a necessary condition for optimal emotion detection. Researchers are now focusing their efforts on developing and commercializing powerful multimodal deep learning architectures and databases, such as the fusion of audio and visual modalities investigated by Zhang et al. [124] and Ringeval et al. [125] for audio-visual and physiological modalities.

## Figures and Tables

**Table 1:** Comparison between FER models.

Approach	Technique	Groups	Sub	Authors	Acc (%)
DCBiLSTM	Fusion	6	123	Liang et al. [111]	99.6
Dist-based	Optical flow	5	8	Essa & pentland [112]	98
CNN	Facial AUs	7	123	Hashemi et al.,	97.01
SBN-CNN	Batch norm	7	10	Wei et al., [113]	96.8
Rule-based	Optical flow	6	32	Yacoob & davis [114]	95
HMM	2-D FT optical flow	6	4	Otsuka & Ohya	93
TRN	Relational reasoning	8	27	Pise et al. [8,115]	92.7
Rule-based	Parametric model	6	40	Black & Yacoob [116]	92
NN	Optical flow	2	32	Rosenblum et al. [117]	88

## Data Availability

The data that support the findings of this study are available on request from the corresponding author.

## References

[1] Aleksic P. S, Katsaggelos A. K. (2006). Automatic facial expression recognition using facial animation parameters and multistream hmms. *IEEE Transactions on Information Forensics and Security*.

[2] Chouhayebi H., Riffi J., Mahraz M. A., Ali Y., Hamid T. Facial expression recognition using machine learning.

[3] Audumbar Pise Anil., Vadapalli H., Sanders I. (2021). Relational reasoning using neural networks: a survey. *International Journal of Uncertainty, Fuzziness and Knowledge-Based Systems*.

[4] Kim J., Ricci M., Serre T. (2018). Not-So-CLEVR: learning same-different relations strains feedforward neural networks. *Interface focus*.

[5] Sariyanidi E., Gunes H., Cavallaro A. (2014). Automatic analysis of facial affect: a survey of registration, representation, and recognition. *IEEE Transactions on Pattern Analysis and Machine Intelligence*.

[6] Brooks R. (1986). A robust layered control system for a mobile robot. *IEEE Journal of Robotics and Automation*.

[7] Chen C., Li K., Teo S. G., Zou X., Li K., Zeng Z. (2020). Citywide traffic flow prediction based on multiple gated spatio-temporal convolutional neural networks. *ACM Transactions on Knowledge Discovery from Data*.

[8] Pise A., Vadapalli H., Sanders I. (2020). Facial Emotion Recognition Using Temporal Relational Network: An Application to E-Learning. *Multimedia Tools and Applications*.

[9] Rouast P. V., Adam M., Raymond C. (2019). Deep Learning for Human Affect Recognition: Insights and New Developments. *IEEE Transactions on Affective Computing*.

[10] Anne M., Cohn J. F. (2009). *Anatomy of Face*.

[11] Paul E., Matsumoto D., Wallace V. F. (1997). Facial expression in affective disorders. *What the face reveals: Basic and applied studies of spontaneous expression using the Facial Action Coding System (FACS)*.

[12] Kanade T., Cohn J. F., Tian Y. Comprehensive database for facial expression analysis.

[13] Mei J., Li K., Li K. (2017). Customer-satisfaction-aware optimal multiserver configuration for profit maximization in cloud computing. *IEEE Transactions on Sustainable Computing*.

[14] Wallace V. F., Paul E. (1983). Emfacs-7: emotional facial action coding system. *University of California at San Francisco*.

[15] Schubert G. (1990). Human ethology and evolutionary epistemology: the strange case of dmEibesfeldthuman eieeyaPp. xvi, 848, $69.95. *Journal of Social and Biological Systems*.

[16] Jaha E. S., Ghouti L. Color face recognition using quaternion pca.

[17] Ekman P., Di Perrett, Ellis H. D. (1992). Facial expressions of emotion: an old controversy and new findings: Discussion. *Philosophical Transactions of the Royal Society of London,Series A B*.

[18] Zhou X., Li K., Xiao G., Zhou Y., Li K. (2016). Top f probabilistic products queries. *IEEE Transactions on Knowledge and Data Engineering*.

[19] Bichsel M., Pentland A. P. (1994). Human face recognition and the face image Set′s topology. *CVGIP: Image Understanding*.

[20] Li K., Ai W., Tang Z. (2014). Hadoop recognition of biomedical named entity using conditional random fields. *IEEE Transactions on Parallel and Distributed Systems*.

[21] Tang X., Li K., Zeng Z., Veeravalli B. (2010). A novel security-driven scheduling algorithm for precedence-constrained tasks in heterogeneous distributed systems. *IEEE Transactions on Computers*.

[22] Rowley H. A., Baluja S., Kanade T. (1998). Neural network-based face detection. *IEEE Transactions on Pattern Analysis and Machine Intelligence*.

[23] Osuna E., Freund R., Girosi F. Training support vector machines: an application to face detection.

[24] Chen J., Li K., Tang Z. (2016). A parallel random forest algorithm for big data in a spark cloud computing environment. *IEEE Transactions on Parallel and Distributed Systems*.

[25] Viola P., Jones M. Rapid object detection using a boosted cascade of simple features.

[26] Franklin C. Crow. Summed-area tables for texture mapping.

[27] Li H., Li K., An J., Li K. (2017). Msgd: a novel matrix factorization approach for large-scale collaborative filtering recommender systems on gpus. *IEEE Transactions on Parallel and Distributed Systems*.

[28] Liu F., Lin X., Li S. Z., Shi Y. Multi-modal face tracking using bayesian network.

[29] Li S. Z, Zhenqiu Zhang Z. Q. (2004). Floatboost learning and statistical face detection. *IEEE Transactions on Pattern Analysis and Machine Intelligence*.

[30] Xu Y., Li K., He L., Zhang L., Li K. (2014). A hybrid chemical reaction optimization scheme for task scheduling on heterogeneous computing systems. *IEEE Transactions on Parallel and Distributed Systems*.

[31] Gu H., Su G., Cheng Du (2003). Feature points extraction from faces. *Image and vision computing NZ*.

[32] Fasel I. R., Bartlett M. S., Movellan J. R. A comparison of gabor filter methods for automatic detection of facial landmarks.

[33] Cootes T. F., Taylor C. J., Cooper D. H., Graham J. (1995). Active shape models-their training and application. *Computer Vision and Image Understanding*.

[34] Lanitis A., Taylor C. J., Cootes T. F. (1997). Automatic interpretation and coding of face images using flexible models. *IEEE Transactions on Pattern Analysis and Machine Intelligence*.

[35] Cootes T. F., Taylor C. J., Lanitis A. Multi-resolution search with active shape models.

[36] Jiao F., Li S., Shum Heung-Yeung., Dale S. Face alignment using statistical models and wavelet features.

[37] Stan Z., Zhang H. J., Cheng Q. S. Multi-view face alignment using direct appearance models.

[38] Chen Y., Li K., Yang W., Xiao G., Xie X., Li T. (2018). Performance-aware model for sparse matrix-matrix multiplication on the sunway taihulight supercomputer. *IEEE Transactions on Parallel and Distributed Systems*.

[39] Xue R., Yu S., Zhang X. (2020). Identification of parameters in 2d-fem of valve piping system within npp utilizing seismic response. *Computers, Materials & Continua*.

[40] Vasilescu M. A. O., Terzopoulos D. (2002). Multilinear analysis of image ensembles: Tensorfaces. *European Conference on Computer Vision*.

[41] Cootes T. F., Edwards G. J., Taylor C. J. (2001). Active appearance models. *IEEE Transactions on Pattern Analysis and Machine Intelligence*.

[42] Kimura S., Yachida M. Facial expression recognition and its degree estimation.

[43] Colmenarez A., Frey B., Huang T. S. A probabilistic framework for embedded face and facial expression recognition.

[44] De Valois R. L., De Valois K. K. (1980). Spatial vision. *Annual Review of Psychology*.

[45] Jones J. P., Palmer L. A. (1987). An evaluation of the two-dimensional gabor filter model of simple receptive fields in cat striate cortex. *Journal of Neurophysiology*.

[46] Xie Y., Hu L., Chen X., Feng J., Zhang D. (2020). Auxiliary diagnosis based on the knowledge graph of tcm syndrome. *Computers, Materials & Continua*.

[47] Yu J., Bhanu B. (2006). Evolutionary feature synthesis for facial expression recognition. *Pattern Recognition Letters*.

[48] Arpaci I., Alshehabi S., Al-Emran M. (2020). Analysis of twitter data using evolutionary clustering during the covid-19 pandemic. *Computers, Materials & Continua*.

[49] Matsuno K., Lee C.-W., Kimura S., Tsuji S. Automatic recognition of human facial expressions.

[50] Shinohara Y., Otsuf N. 1 Facial expression recognition using Fisher weight maps.

[51] Wu Yu-K., Lai S.-H. Facial expression recognition based on supervised lle analysis of optical flow and ratio image.

[52] Wang H. Facial expression decomposition.

[53] Yin L., Wei X. Multi-scale primal feature based facial expression modeling and identification.

[54] Kotsia I., Pitas I. (2006). Facial expression recognition in image sequences using geometric deformation features and support vector machines. *IEEE Transactions on Image Processing*.

[55] Xu Q., Zhang P., Pei W., Yang L., He Z. A facial expression recognition approach based on confusion-crossed support vector machine tree.

[56] Michel P., El Kaliouby R. Real time facial expression recognition in video using support vector machines.

[57] Bartlett M. S., Littlewort G., Fasel I., Movellan J. R. Real time face detection and facial expression recognition: development and applications to human computer interaction.

[58] Ma L., Khorasani K. (2004). Facial expression recognition using constructive feedforward neural networks. *IEEE Transactions on Systems, Man and Cybernetics, Part B (Cybernetics)*.

[59] Zhang Z., Lyons M., Schuster M., Akamatsu S. Comparison between geometry-based and gabor-wavelets-based facial expression recognition using multi-layer perceptron.

[60] Kobayashi H., Tange A., Hara F. (1996). Space robot. Real-time recognition of 6 basic facial expressions. *Journal of the robotics society of Japan*.

[61] Ichimura T., Oeda S., Yamashita T. Construction of emotional space from facial expression by parallel sand glass type neural networks.

[62] Picard R. W., Papert S., Bender W. (2004). Affective learning - a manifesto. *BT Technology Journal*.

[63] Rafiq M., Ahmadian A., Raza A., Baleanu D., Sarwar Ahsan M., Hasan Abdul Sathar M. (2020). Numerical control measures of stochastic malaria epidemic model. *Computers, Materials & Continua*.

[64] Moridis C. N., Economides A. A. (2008). Toward computer-aided affective learning systems: a literature review. *Journal of Educational Computing Research*.

[65] Tan Y., Qin J., Xiang X., Zhang C., Wang Z. (2021). Coverless Steganography Based on Motion Analysis of Video. *Security and Communication Networks*.

[66] Yuan N., Jia C., Lu J. (2020). A drl-based container placement scheme with auxiliary tasks. *Computers, Materials & Continua*.

[67] Yan B., Wang J., Zhang Z. (2020). An improved method for the fitting and prediction of the number of covid-19 confirmed cases based on lstm. *Computers, Materials & Continua*.

[68] Bartlett M. S., Littlewort G., Frank M., Lainscsek C., Fasel I., Movellan J. Recognizing facial expression: machine learning and application to spontaneous behavior.

[69] Mj Den Uyl, Van Kuilenburg H. (2005). The facereader: online facial expression recognition. *Proceedings of the measuring behavior*.

[70] Happy S. L., George A., Routray A. A real time facial expression classification system using local binary patterns.

[71] shou Xie P., qiang Ma G., Feng T., Yan Y., ming Han X. (2020). Behavioral feature and correlative detection of multiple types of node in the internet of vehicles. *Computers, Materials & Continua*.

[72] Ao F., Gao Z., Song X., Ke Ke, Xu T., Zhang X. (2020). Modeling multi-targets sentiment classification via graph convolutional networks and auxiliary relation. *Computers, Materials & Continua*.

[73] Shiah Y. C., Huang S.-C., Hematiyan M. R. (2020). Efficient 2d analysis of interfacial thermoelastic stresses in multiply bonded anisotropic composites with thin adhesives. *Computers, Materials & Continua*.

[74] Zhang S., Zhao X., Lei B. (2012). Facial expression recognition based on local binary patterns and local Fisher discriminant analysis. *WSEAS transactions on signal processing*.

[75] Zhou S., Ke M., Luo P. (2019). Multi-camera transfer gan for person re-identification. *Journal of Visual Communication and Image Representation*.

[76] Fei Yu, Tang Q., Wang W., Wu H. (2016). A 2.7 ghz low-phase-noise lc-qvco using the gate-modulated coupling technique. *Wireless Personal Communications*.

[77] Long M., Zeng Y. (2019). Detecting iris liveness with batch normalized convolutional neural network. *Computers, Materials & Continua*.

[78] Lucey P., Cohn J. F., Kanade T., Saragih J., Ambadar Z., Matthews I. The extended cohn-kanade dataset (ck+): a complete dataset for action unit and emotion-specified expression.

[79] Liu L., Özsu M. T. (2009). *Encyclopedia of Database Systems*.

[80] Daneshmand M., Abels A., Anbarjafari G. (2017). Real-time, automatic digi-tailor mannequin robot adjustment based on human body classification through supervised learning. *International Journal of Advanced Robotic Systems*.

[81] Bolotnikova A., Demirel H., Anbarjafari G. (2017). Real-time ensemble based face recognition system for nao humanoids using local binary pattern. *Analog Integrated Circuits and Signal Processing*.

[82] Valstar M., Schuller B., Smith K. Avec 2013: the continuous audio/visual emotion and depression recognition challenge.

[83] Watson C., Flanagan P. (2016). Nist special database 18 mugshot identification database,.

[84] Bruce V., Young A. (1986). Understanding face recognition. *British Journal of Psychology*.

[85] Richard G., Mengay Y., Guis I. Multi modal verification for teleservices and security applications (m2vts).

[86] Paul E. (1999). Basic emotions. *Handbook of cognition and emotion*.

[87] Oppermann R., Rasher R. (1997). Adaptability and adaptivity in learning systems. *Knowledge transfer*.

[88] Popescu E. (2009). Diagnosing students’ learning style in an educational hypermedia system. *Cognitive and Emotional Processes in Web-Based Education: Integrating Human Factors and Personalization*.

[89] Prakash D., Van Haneghan J., Blackwell W., Jackson S., Murugesan G., Tamilselvan K. S., Alam M. S. (1995). Classroom engagement evaluation using computer vision techniques. *Pattern Recognition and Tracking XXX*.

[90] Li J., Wang P., Xu Y. (2017). Prognostic value of programmed cell death ligand 1 expression in patients with head and neck cancer: a systematic review and meta-analysis. *PLoS One*.

[91] Aung M. S. H., Alquaddoomi F., Hsieh C.-K. (2016). Leveraging multi-modal sensing for mobile health: a case review in chronic pain. *IEEE journal of selected topics in signal processing*.

[92] O’regan K. (2003). Emotion and e-learning. *Journal of Asynchronous Learning Networks*.

[93] Kemp N., Grieve R. (2014). Face-to-face or face-to-screen? Undergraduates’ opinions and test performance in classroom vs. online learning. *Frontiers in Psychology*.

[94] Xu Q., Sun Bo, He J., Rong B., Yu L., Rao P. Multimodal facial expression recognition based on dempster-shafer theory fusion strategy.

[95] Yao J. (2015). Multilayer model for on-line learning resources based on cognitive load theory. *World Trans. on Engng. and Technol. Educ*.

[96] Joseph R., Divvala S., Girshick R., Ali F. You only look once: unified, real-time object detection.

[97] Sun X., Lichtenauer J., Valstar M., Nijholt A., Pantic M. A multimodal database for mimicry analysis.

[98] Sivaraman K., Murthy A. Object recognition under lighting variations using pre-trained networks.

[99] Shao J., Qian Y. (2019). Three convolutional neural network models for facial expression recognition in the wild. *Neurocomputing*.

[100] Stoyanov S., Kirchner P. (2004). Expert concept mapping method for defining the characteristics of adaptive e-learning: alfanet project case. *Educational Technology Research & Development*.

[101] Ren S., He K., Girshick R., Sun J. (2017). Faster r-cnn: towards real-time object detection with region proposal networks. *IEEE Transactions on Pattern Analysis and Machine Intelligence*.

[102] Varga D. (2020). Multi-pooled inception features for no-reference image quality assessment. *Applied Sciences*.

[103] Krizhevsky A., Sutskever I., Hinton G. E. (2012). Imagenet classification with deep convolutional neural networks. *Advances in Neural Information Processing Systems*.

[104] Tomè D., Monti F., Baroffio L., Bondi L., Tagliasacchi M., Tubaro S. (2015). Deep convolutional neural networks for pedestrian detection. *CoRR, abs/*.

[105] Lopes A. T., de Aguiar E., De Souza A. F., Oliveira-Santos T., Oliveira-Santos T. (2017). Facial expression recognition with Convolutional Neural Networks: c. *Pattern Recognition*.

[106] Cai J., Chang O., Tang X. L., Xue C., Wei C. Facial expression recognition method based on sparse batch normalization cnn.

[107] Li Y., Zeng J., Shan S., Chen X. (2019). Occlusion aware facial expression recognition using CNN with attention mechanism. *IEEE Transactions on Image Processing*.

[108] Yolcu G., Oztel I., Kazan S. (2019). Facial expression recognition for monitoring neurological disorders based on convolutional neural network. *Multimedia Tools and Applications*.

[109] Agrawal A., Mittal N. (2020). Using cnn for facial expression recognition: a study of the effects of kernel size and number of filters on accuracy. *The Visual Computer*.

[110] Kumar Jain Deepak., Shamsolmoali P., Sehdev P. (2019). Extended deep neural network for facial emotion recognition. *Pattern Recognition Letters*.

[111] Liang D., Liang H., Yu Z., Zhang Y. (2020). Deep convolutional bilstm fusion network for facial expression recognition. *The Visual Computer*.

[112] Essa I.A., Pentland A., Trevor Darrell Tracking facial motion.

[113] Wei C., Cai J., Chang O., Tang X. -L., Xue C. Facial expression recognition method based on sparse batch normalization cnn.

[114] Yacoob Y., Davis L. S. (1996). Recognizing human facial expressions from long image sequences using optical flow. *IEEE Transactions on Pattern Analysis and Machine Intelligence*.

[115] Audumbar Pise Anil., Vadapalli Hima., Sanders I. (2022). Estimation of learning affects experienced by learners: an approach using relational reasoning and adaptive mapping. *Wireless Communications and Mobile Computing*.

[116] Black M. J., Yacoob Y. (1997). Recognizing facial expressions in image sequences using local parameterized models of image motion. *International Journal of Computer Vision*.

[117] Rosenblum H. J., Pace-Savitsky K., Perry R. J., Kramer J. H., Miller B. L., Levenson R. W. (2004). Recognition of emotion in the frontal and temporal variants of frontotemporal dementia. *Dementia and Geriatric Cognitive Disorders*.

[118] Kim D. H., Wissam J., Jang J., Man Ro Y. (2017). Multi-objective based spatio-temporal feature representation learning robust to expression intensity variations for facial expression recognition. *IEEE Transactions on Affective Computing*.

[119] Yu Z., Liu G., Liu Q., Deng J. (2018). Spatio-temporal convolutional features with nested lstm for facial expression recognition. *Neurocomputing*.

[120] Lopes A. T., de Aguiar E., De Souza A. F., Santos T. O. (2017). Facial expression recognition with convolutional neural networks: coping with few data and the training sample order. *Pattern Recognition*.

[121] Ali M., Chan D., Mahoor M. H. Going deeper in facial expression recognition using deep neural networks.

[122] Mohammadpour M., Khaliliardali H., R Hashemi S. M., AlyanNezhadi M. M. Facial emotion recognition using deep convolutional networks.

[123] Pantic M., Rothkrantz L. J, Rothkrantz M. (2003). Toward an affect-sensitive multimodal human-computer interaction. *Proceedings of the the IEEE*.

[124] Zhang S., Zhang S., Huang T., Gao W. Multimodal deep convolutional neural network for audio-visual emotion recognition.

[125] Ringeval F., Eyben F., Kroupi E. (2015). Prediction of asynchronous dimensional emotion ratings from audiovisual and physiological data. *Pattern Recognition Letters*.

